# Immunohistochemical biomarkers and volumetric parameters for predicting radiotherapy-based outcomes in patients with p16-negative pharyngeal cancer

**DOI:** 10.18632/oncotarget.20374

**Published:** 2017-08-21

**Authors:** Rui-Yun Chen, Ying-Chun Lin, Shang-Wen Chen, Tze-Yi Lin, Te-Chun Hsieh, Kuo-Yang Yen, Ji-An Liang, Shih-Neng Yang, Yao-Ching Wang, Ya-Huey Chen, Shu-Fen Chiang, Chia-Hung Kao

**Affiliations:** ^1^ Department of Pathology, China Medical University Hospital, Taichung, Taiwan; ^2^ Department of Radiation Oncology, China Medical University Hospital, Taichung, Taiwan; ^3^ The Ph.D. Program for Cancer Biology and Drug Discovery, China Medical University and Academia Sinica, Taichung, Taiwan; ^4^ School of Medicine, China Medical University, Taichung, Taiwan; ^5^ School of Medicine, Taipei Medical University, Taipei, Taiwan; ^6^ Graduate Institute of Clinical Medical Science, School of Medicine, College of Medicine, China Medical University, Taichung, Taiwan; ^7^ Department of Nuclear Medicine and PET Center, China Medical University Hospital, Taichung, Taiwan; ^8^ Department of Biomedical Imaging and Radiological Science, China Medical University, Taichung, Taiwan; ^9^ Graduate Institute of Cancer Biology, China Medical University, Taichung, Taiwan; ^10^ Center for Molecular Medicine, China Medical University Hospital, Taichung, Taiwan; ^11^ Cancer Center, China Medical University Hospital, Taichung, Taiwan; ^12^ Department of Bioinformatics and Medical Engineering, Asia University, Taichung, Taiwan

**Keywords:** squamous cell carcinoma, p16-negative pharyngeal cancer, biomarker, positron emission tomography–computed tomography, gross tumor volume

## Abstract

**Background:**

This study determined the prognostic effects of immunohistochemical biomarkers and volumetric parameters predicting radiotherapy-based treatment in patients with p16-negative squamous cell carcinoma of the oropharynx or hypopharynx.

**Results:**

VEGF immunoreactivity > 2 and GLUT1 overexpression were prognostic factors for lower cause-specific survival. Moreover, both factors were associated with lower disease-free survival. The predictors of lower primary relapse-free survival were VEGF immunoreactivity > 2 and CT-based gross tumor volume > 16 mL.

**Materials and Methods:**

Immunohistochemical biomarkers in pretreatment biopsy specimens from 60 patients with p16-negative cancer were analyzed using tissue microarrays. Computed tomography (CT)-based and biological tumor volumes were retrieved through fluorodeoxyglucose positron emission tomography-CT. Correlations of cause-specific, disease-free, and primary relapse-free survival with volumetric parameters and the immunohistochemical biomarker score were investigated.

**Conclusions:**

For patients with p16-negative pharyngeal cancer receiving radiotherapy, treatment outcomes can be stratified by VEGF and GLUT1 expression and CT-based gross tumor volume.

## INTRODUCTION

Human papilloma virus (HPV) infection has become increasingly apparent as a major risk factor for head and neck cancer [1, 2]. HPV-positive tumors are clinically, pathologically, and etiologically distinct and are more responsive to treatment [3, 4]. Therefore, despite their occurrence in the same tissue, evidence suggests a biological distinction between HPV-positive and HPV-negative head and neck cancers [5].

The prevalence of human HPV-positive pharyngeal cancer (PC) is lower than 20% in Asia, which is lower than that in Western countries [6–8]. Although several studies have assessed the ability of various endogenous hypoxic, radioresistant, and proliferative biomarkers to predict treatment outcomes [9–20], few studies have analyzed HPV-negative cancer cohorts. By combining the biological tumor volume determined using fluorine-18 fluorodeoxyglucose (FDG) positron emission tomography–computed tomography (PET-CT) and some immunohistochemical markers, our previous study showed an overexpression of certain endogenous markers associated with increased FDG uptake and treatment outcomes [21]. However, the implementation of precision medicine for HPV-negative PCs remains limited by the lack of thorough information on individual responses to a specific treatment, particularly for patients with advanced stages of cancer. Hence, this study determined the optimal approach for predicting radiotherapy (RT) or chemoradiotherapy (CRT) for organ preservation in patients with HPV-negative PCs by comparing imaging and immunohistochemical studies. The model was based on the assumption that biological and volumetric parameters should be assessed simultaneously to optimize patient selection. In addition, the prognostic effects of biological markers can provide novel therapeutic implications for future clinical trials. The results can facilitate optimizing treatment schemes for HPV-negative patients with high risk factors.

## RESULTS

### Treatment outcomes

Sixty patients were eligible for this study (Table 1). At a median follow-up of 23 months (6–72 mo), 27 patients were alive without any observed disease recurrence, and 33 patients had recurrent disease. Twenty-four patients died of tumor recurrence, and 5 died of other malignancies. Supplementary Appendix 1 shows the detailed patterns of treatment failure in the study cohort. In summary, 31 patients remained relapse-free at the primary sites, whereas 29 patients had primary recurrence. Overall, the 2-year CSS, DFS, and PRFS rates were 53% (95% confidence interval [CI] = 41%–66%), 50% (95% CI = 38%–63%), and 49% (95% CI = 37%–62%), respectively. The survival curves according to AJCC T- and N-classification are illustrated in Supplementary Appendix 2.

**Table 1:** Characteristics of p16-negative patients (*N* = 60)

Variables	*N* (%)
Sex	
Male	60
Age (year)	range 27 to 78 (median 53)
Primary tumor site	
oropharynx	33 (55%)
hypopharynx	7 (45%)
T stage	
T1	3 (5%)
T2	23 (38%)
T3	18 (30%)
T4	16 (27%)
N stage	
N1	8 (13%)
N2	48 (80%)
N3	4 (7%)
AJCC classification (7th version)	
III	4 (7%)
IVA	51 (85%)
IVB	5 (8% )
Histology grade of squamous cell carcinoma	
well differentiated	20 (33%)
moderately differentiated	17 (28%)
poorly differentiated	10 (17%)
unclassified	13 (22%)
Smoking	
smoker	56 (93%)
never-smoker	4 (7%)
Betel nut quid	
yes	48 (80%)
never	12 (20%)
Alcohol drinking	
yes	43 (72%)
never	17 (28%)
Radiation dose (Gy)	median 70 Gy (range, 66 - 74Gy)
Concurrent drug regimen	
cisplatin every 3 weeks	47 (78%)
cetuximab	10 (17%)
none	3 (5%)
Median follow-up durations (months)	23 (range, 6 to 72)

Abbreviations: AJCC = the American Joint Committee on Cancer.

### Comparison of the predictive ability of different threshold methods for local failure

The ROC curves were analyzed to compare the efficacy of various PET-CT-related parameters and threshold methods for determining the optimal approach for autosegmentation contouring. The results revealed that MTV2.5, TLGp40%, TLGw40% were stronger predictors of a residual or recurrent tumor than were other corresponding threshold methods (Supplementary Appendix 3). According to the results, biological tumor volumes determined using the MTV2.5, TLGp40%, and TLGw40% methods combined with GTVp and the optimal cutoff of the immunohistochemistry scoring system for different biomarker expressions (Supplementary Appendix 4) were selected for analyses.

### Correlation between volumetric parameters and immunohistochemical biomarker expression

For all patients, MTV and TLG values were calculated using 4 methods. We failed to obtain comprehensive data on immunohistochemical biomarker expression for 3 patients. *GLUT1* overexpression was positively associated with increase of SUVmax and TLG values. *VEGF* IRS > 2 was correlated with MTV2.5 and TLGw40%. *c-Met* overexpression was correlated with higher MTV2.5 and TLG values (Table 2). Furthermore, higher GTVp was correlated with the overexpression of *HIF-1α* (*P* = .02, γ = 0.31), *VEGF* (*P* = .01, γ = 0.34), and *c-Met* (*P* = .02, γ = 0.30). Typically, the results revealed weak associations between volumetric data and immunohistochemical biomarkers.

**Table 2:** Correlation between PET-CT parameters and protein biomarkers

Variables	SUVmax	MTV2.5	TLGp40%	TLGw40%
HIF-1α				
*P* value	0.22	0.13	0.11	0.74
VEGF				
*P* value	0.30	0.003 (γ = 0.38)	0.06	< 0.001 (γ = 0.44)
GLUT1				
*P* value	0.01 (γ = 0.33) 0.12		0.049 (γ = 0.26)	0.01 (γ = 0.31)
CAIX				
*P* value	0.25	0.57	0.39	0.69
CLAUDIN-4				
*P* value	0.15	0.80	0.72	0.43
c-Met				
*P* value	0.10	0.02 (γ = 0.31)	0.02 (γ = 0.32)	0.03 (γ = 0.28)
Bcl-2				
*P* value	0.98	0.49	0.46	0.40
YAP-1				
*P* value	0.57	0.43	0.95	0.81
Ki-67				
*P* value	0.06	0.63	0.52	0.74
EGFR				
*P* value	0.053	0.62	0.30	0.78

### Prognostic factors for CSS, DFS, and PRFS

**Figure 1:**
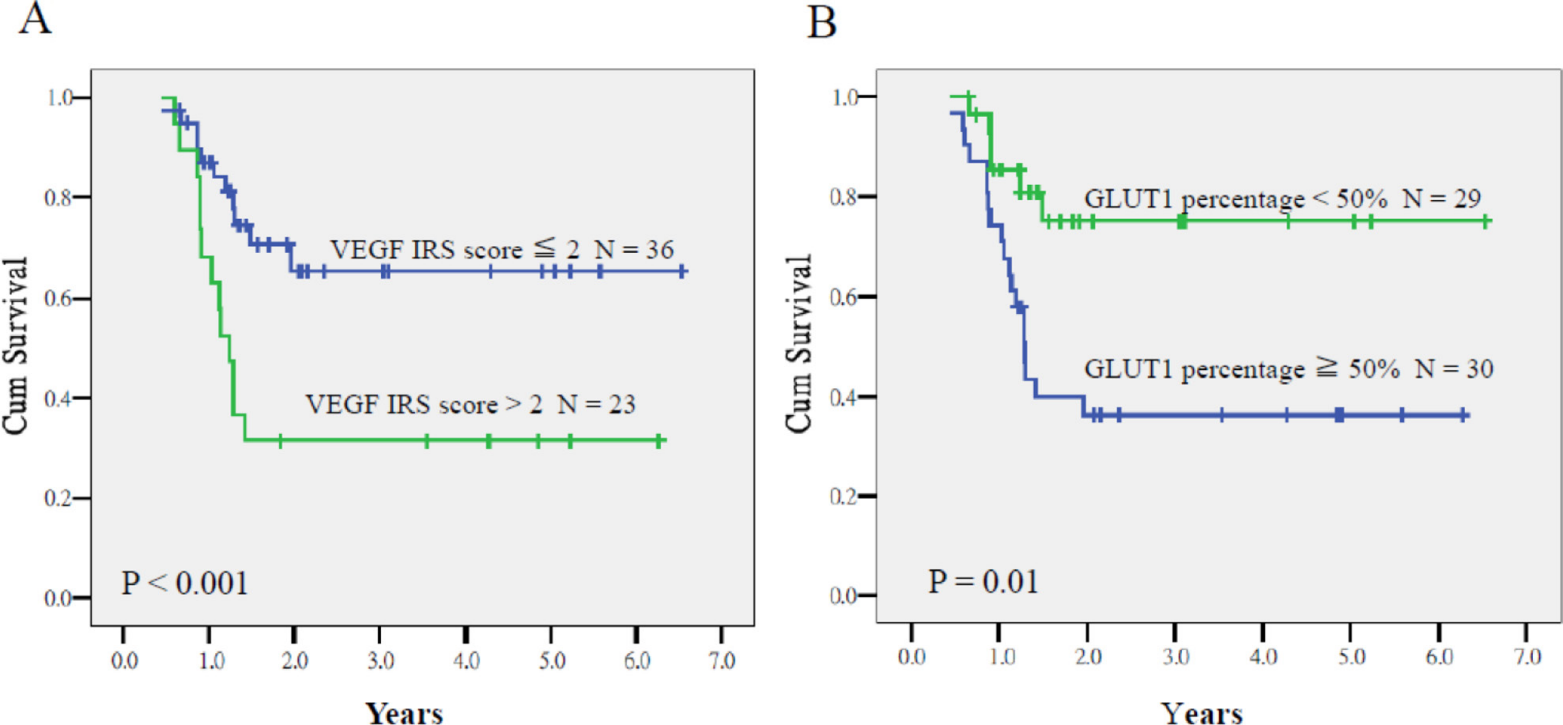
Cause-specific survival of patients who had tumors with *VEGF* IRS > 2 and ≤ 2 (**A**) and with ≥ 90% and < 90% expression of *GLUT1* (**B**) *P* < 0.001 and 0.01, respectively).

**Figure 2:**
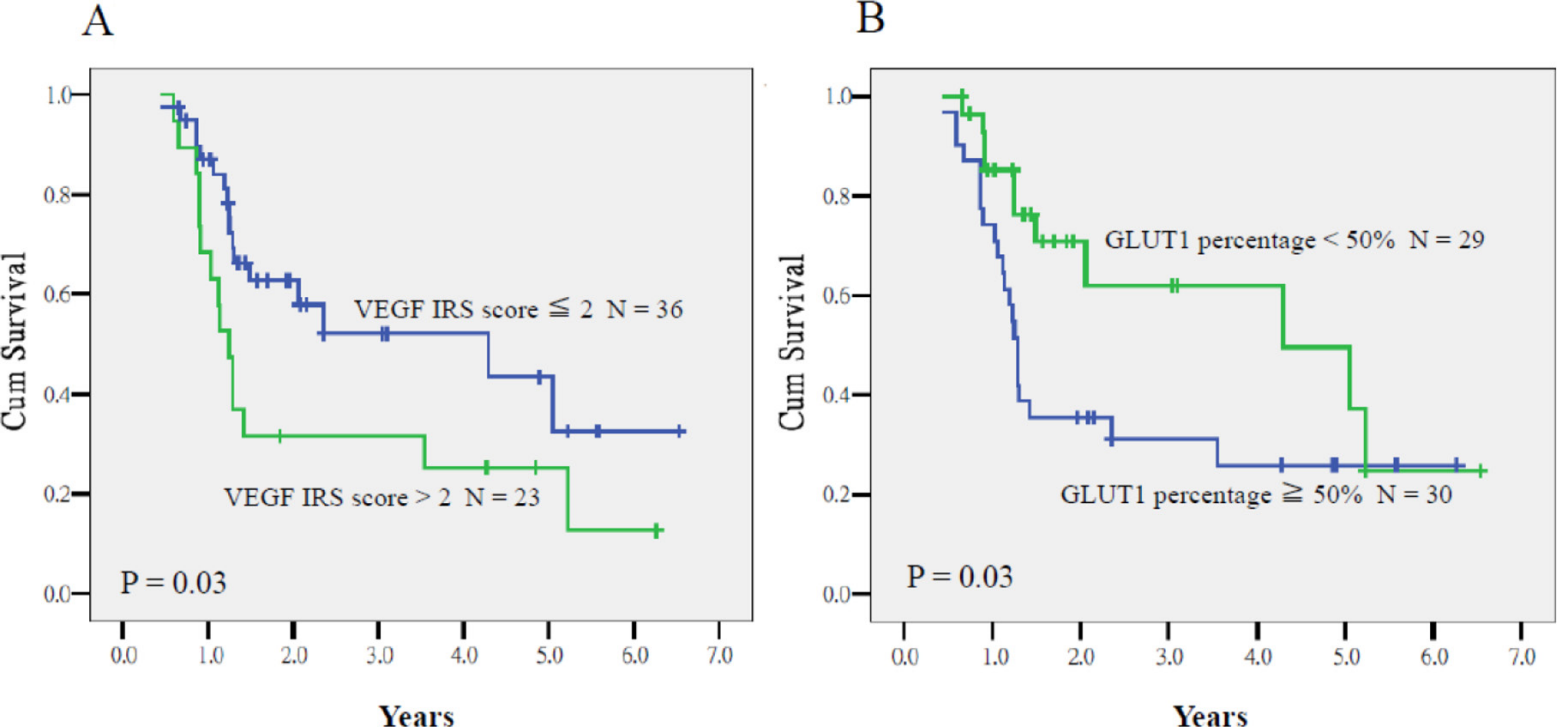
Disease-free survival of patients who had tumors with *VEGF* IRS > 2 and ≤ 2 (**A**) and with ≥ 90% and < 90% expression of *GLUT1* (**B**) (*P* = 0.03 and 0.03, respectively).

As summarized in Table 3, multivariate analyses revealed *VEGF* IRS > 2 [*P* < .001, hazard ratio (HR) = 11.21, 95% CI = 4.96–55.98] and *GLUT1* overexpression (*P* < .001, HR = 13.51, 95% CI = 3.67–47.62) as prognostic factors for lower CSS. The 2-year CSS rates of patients who had tumors with *VEGF* immunoreactive score (IRS) > 2 and ≤ 2 were 32% and 66% (*P* < .001), respectively, whereas the corresponding rates of patients with ≥ 90% and < 90% expression of *GLUT1* were 37% and 75% (*P* = .01 Figure 1). Both prognostic factors were also associated with lower DFS (*P* < .001, HR = 5.18, 95% CI = 2.26–11.83 and *P* = .006, HR = 3.21, 95% CI = 1.40–7.53). The 2-year DFS rates of patients who had tumors with *VEGF* IRS > 2 and ≤ 2 were 32% and 58% (*P* = .03), respectively, whereas the corresponding rates of patients with ≥ 90% and < 90% expression of *GLUT1* were 35% and 62% (*P* = .03; Figure 2). Expression of *HIF-1α* ≥ 80% was the third molecular marker of lower CSS (*P* = .003, HR = 4.95, 95% CI = 1.70–14.49).

**Table 3:** Multivariate analysis using the Cox regression model for overall survival, disease-free survival, and primary relapse-free survival among volumetric parameters or protein biomarkers

Variables	CSS HR 95% CI *P*	DFS HR 95% CI *P*	PRFS HR 95% CI *P*
**TNM classification**			
T stage			
T1–2 vs. T3–4	2.68 0.89–8.64 0.10	3.33 0.41–36.88 0.23	3.19 0.52–23.26 0.26
N-stage			
N1 vs. N2–3	4.15 0.20–81.02 0.36	2.60 0.16– 41.39 0.49	
**Primary tumor volume**			
GTVp (ml)			
< 16 vs. ≧ 16	3.70 0.83–10.47 0.10	3.62 0.79–17.35 0.15	5.52 1.85–16.47 0.002
**PET-CT parameters**			
SUVmax			
< 10.3 vs. ≧10.3	1.68 0.22–13.19 0.62	2.30 0.25–24.50 0.43	2.21 0.36–13.70 0.39
MTV2.5 (ml)			
< 14.5 vs. ≧14.5	8.25 0.59–36.69 0.09	9.80 0.71–82.18 0.09	11.36 0.74–86.67 0.08
TLGp40% (g)			
< 62.4 vs. ≧62.4	3.11 0.56–12.17 0.12	2.99 0.37–17.13 0.76	4.38 0.76–26.32 0.11
TLGw40% (g)			
< 132.5 vs. ≧132.5	4.34 0.21–53.4 0.34	3.89 0.42–18.66 0.39	1.15 0.13–8.38 0.87
**Primary tumor origin**			
oropharynx vs. hypopharynx	2.51 0.46– 12.51 0.29	1.66 0.34–8.20 0.53	2.04 0.41–10.42 0.39
**Immunohistochemistry**			
*GLUT1* stain percentage			
< 90% vs. ≥ 90%	13.51 3.67–47.62 < 0.001	3.21 1.40–7.53 0.006	3.27 0.72–10.17 0.08
*VEGF IRS*			
≦2 vs. > 2	11.21 4.96 −55.98 < 0.001	5.18 2.26–11.83 0.001	2.65 1.46–6.10 0.02
*HIF-1α* stain percentage			
< 80% vs. ≥ 80%	4.95 1.70–14.49 0.003	4.57 0.42–39.56 0.16	6.53 0.56–47.97 0.15
*CAIX* stain percentage			
≦10% vs. > 10%	2.40 0.12–76.92 0.47	3.49 0.22–39.17 0.36	5.05 0.21–99.81 0.32
*EGFR* stain percentage			
< 65% vs. ≥ 65%	1.35 0.20–2.71 0.65	1.23 0.29–4.64 0.89	2.93 0.89–9.55 0.15
*Ki-67*			
<15% vs. ≥ 15%	2.37 0.68–10.76 0.15	2.55 0.72–13.54 0.07	4.32 0.68–27.78 0.12
*Bcl-2* stain			
< 10% vs. ≥ 10%	1.12 0.11–8.53 0.34	1.41 0.09–17.64 0.52	1.71 0.32–9.07 0.53
*CLAUDIN-4* stain percentage			
score 0–4 vs. 5–12	1.23 0.26–5.55 0.80	1.82 0.48–7.76 0.35	1.98 0.56–7.01 0.29
*YAP-1* stain percentage			
< 50% vs. ≥ 50%	2.47 0.44–7.75 0.41	2.08 0.15–29.2 0.58	1.73 0.29–10.43 0.55
*c-Met* stain intensity			
≦20% vs. > 20%	1.73 0.35–8.64 0.46	2.07 0.43–15.92 0.27	2.05 0.78–9.04 0.19

Abbreviations: CSS = cause-specific survival; DFS = disease-free survival; PRFS = primary relapse-free survival; HR = hazard ratio; CI = confidence interval; GTVp = tumor volume at primary site; SUVmax = maximum standard uptake value; MTV2.5 = pretreatment primary metabolic tumor volume defined by SUV = 2.5; TLGp40% = pretreatment primary lesion glycolysis defined by 40% of maximal SUV TLGw40%: pretreatment primary total body glycolysis defined by 40% of maximal SUV; IRS = immunoreactive score.

**Figure 3:**
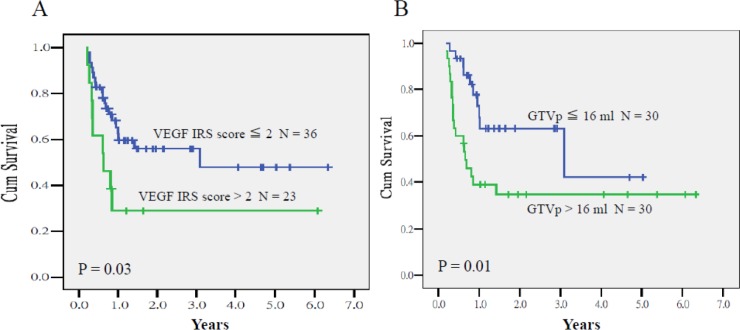
Primary relapse-free survival of patients who had tumors with *VEGF* IRS > 2 and ≤ 2 (**A**) and GTV > 16 and ≤ 16 mL (**B**) *P* = 0.03 and 0.01, respectively).

The predictors of lower PRFS were *VEGF* IRS > 2 (*P* = .02, HR = 2.65, 95% CI = 1.46–6.10) and GTVp > 16 mL (*P* = .002, HR = 5.52, 95% CI = 1.85–16.47). The 2-year PRFS rates of patients who had tumors with *VEGF* IRS > 2 and ≤ 2 were 30% and 56% (*P* = .03), respectively, whereas the corresponding rates of patients with GTVp > 16 and ≤ 16 mL were 35% and 64% (*P* = .01; Figure 3). In addition, no significant difference was observed in PRFS curves between the 2 origin sites of the primary tumors.

To investigate the effects of biological differences on outcomes between the 2 tumor origin sites, subgroup analyses were performed (Supplementary Appendix 5). *VEGF* IRS > 2 remained the major determinant of CSS and DFS after stratification for the tumor origin site. *HIF-1α* and GTVp expression was a prognostic factor for PRFS in patients with hypopharyngeal cancer.

## DISCUSSION

An understanding of cancer cell phenotypes from genomic expression, immunohistochemical studies, or imaging studies allows oncologists to use individualized therapy. To date, no multigenomic assay is available for clinical practice in patients with p16-negative PCs. This study is the first to compare comprehensive quantitative immunohistochemical biomarkers and volumetric parameters for predicting the outcomes in patients with advanced p16-negative PCs receiving definitive RT-based treatment. The assessment of various biomarkers revealed 2 endogenous hypoxic markers, *VEGF* IRS > 2 and *GLUT1* overexpression, as major prognostic factors for lower CSS and DFS. In particular, *VEGF* expression maintained its predictive ability per site. In addition, *VEGF* IRS > 2 and higher GTVp were predictors of lower local control. On the basis of our finding, certain biological characteristics of tumors might be more determinant than the volumetric parameters alone for RT-based treatment. Moreover, clinical trials with a novel therapeutic strategy should be considered for patients with adverse features.

The most valuable finding is that compared with various biomarkers or volumetric methods, tumor hypoxia remained a major cause of treatment failure in patients with p16-negative PCs because *GLUT1*, *VEGF*, and *HIF-1α* were found to be associated with inferior outcomes. Several studies on head and neck cancers have concluded tumor hypoxia as a major determinant of treatment outcomes [22]. Because endogenous markers can indicate therapeutically relevant levels of hypoxia within tumors, clinical trials assessing the ability of a marker to predict the benefit of specific hypoxia-directed treatment are warranted. Moreover, this study is the first to disclose that *GLUT1* overexpression contributed to inferior RT-based outcome in patients with p16-negative PCs. In agreement with a stratified systemic analysis [23], expression status of *GLUT1* was associated with unfavorable clinical results of oral squamous cell carcinoma (Odds ratio = 3.79; 95% CI = 1.74–8.24, *P* = 0.0008). In addition, our study examined two characters associated with the glycolytic phenotypes of cancers: *GLUT1* expression level and FDG uptake on PET-CT. Although *GLUT1* expression was positively associated with increased SUVmax and TLG values, *GLUT1* overexpression influenced the treatment outcome more straightforward.

Many studies have confirmed the prognostic importance of angiogenesis markers and have found an association of these markers with the progression of tumors and development of lymph node metastases. In accordance with a meta-analysis [16], our study highlighted the prognostic effects of *VEGF* on RT-based treatment. Accordingly, a combination of anti-*VEGF* target therapies should be used for treating patients with PCs exhibiting *VEGF* overexpression. Two clinical trials have reported the safety and feasibility of the incorporation of bevacizumab into comprehensive CRT regimens for patients with head and neck cancers [24, 25]. Further studies must test this regimen in an appropriate subset of patients receiving CRT, particularly those with p16-negative PCs.

In this study, GTVp was the only volumetric parameter affecting local control. GTVp was related to *HIF-1α*, *VEGF*, and *c-Met* overexpression. Although we failed to demonstrate the effects of FDG uptake on several endpoints, the PET-related volumetric parameters were clearly associated with certain genomic expression patterns. In particular, the overexpression of *GLUT1*, *VEGF*, *HIF-1α*, and *c-Met* was positively correlated with tumors with an increased FDG uptake, in concordance with previous studies [26, 27]. The predictive ability of endogenous molecular markers was more satisfactory than that of volumetric parameters. Endogenous biomarkers were the major determinants of the final outcomes; therefore, it is imperative to understand the intrinsic biological characteristics of tumors rather than indiscriminately increasing the RT or drug dose.

The present findings should be cautiously interpreted because of the limited sample size and retrospective design of the study. Therefore, the multivariate analysis per site should be strengthened by conducting additional validation studies by using a large patient cohort. Moreover, RT and CRT outcomes should be assessed with many other biomarkers, such as DNA repair genes, which were not included in the scope of this study. However, the strengths of this study include the uniform treatment strategies and comprehensive quantitative immunohistochemistry methods. Although our findings support the clinical evaluation of hypoxic sensitizers in patients with p16-negative locally advanced PCs, a limitation is the lack of ^18^F-labeled nitroimidazoles PET-CT for imaging the hypoxia for comparison. Because hypoxia presents both treatment challenges and opportunities, characterization is essential to determine the most effective therapy [22]. Considering the more favorable response to conventional RT in patients with p16-positive tumors, the major benefit of hypoxic modification may be improved outcomes in patients with p16-negative tumors [28]. Hence, an appropriate therapy can be decided by evaluating individual tumor biology and assessing established prognostic and predictive biomarkers.

## MATERIALS AND METHODS

### Study population

**Figure 4:**
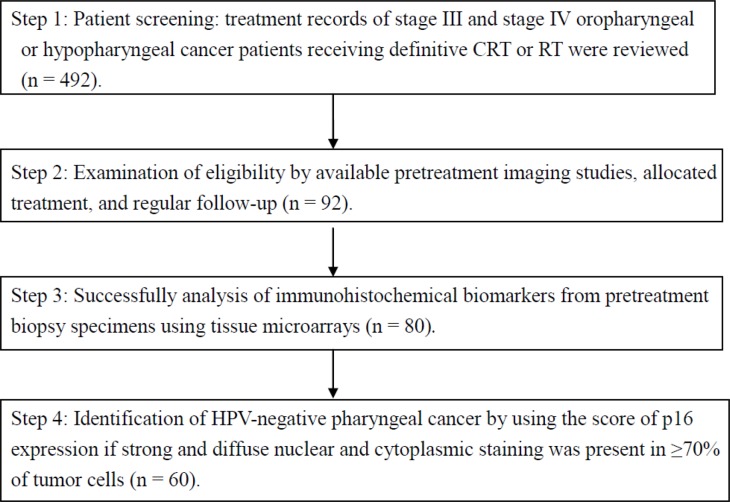
Flowchart of patient selection and study design

This retrospective cohort study recruited 92 patients with newly diagnosed squamous cell carcinoma of the oropharynx or hypopharynx who were scheduled to undergo definitive CRT or RT at China Medical University Hospital between January 2007 and December 2013. These patients were selected because they had received RT-based treatment. All patients had received pretreatment PET-CT for RT planning or pretreatment staging; each had a normal serum glucose level before undergoing PET-CT. Figure 4 presents the flowchart of patient selection and study design. This study was approved by a local institutional review board (CMUH103-REC2-093FR and DMR99-IRB-010-1).

### Immunohistochemistry

Protein biomarkers in pretreatment incisional biopsy specimens from the primary sites, including endogenous hypoxic markers (*GLUT1*, *CAIX*, *VEGF*, and *HIF-1α*) [18], radioresistant biomarkers *(Bcl-2*, *CLAUDIN-4*, *YAP-1*, and *c-Met*) [9], a proliferative marker (*Ki-67*) [20], and a tumor progression factor (*EGFR*) [13, 19], were analyzed using tissue microarrays. Particularly, *GLUT1* expression in tumors was reported as a glycolytic phenotype [23, 29], and is transcriptionally activated by hypoxia and *HIF-1α* in glucose metabolism [30]. Triplicate 0.6-mm cores were obtained from each paraffin-embedded tumor tissue block. Furthermore, 4-μm-thick paraffin sections were deparaffinized, microwaved according to standard procedures, and processed for immunohistochemical staining. Among the 92 patients, molecular biomarkers were successfully analyzed in 80 blocks.

The slides were scored by 2 pathologists blinded to clinical outcomes. The staining was graded using the immunoreactive score (IRS) system based on the intensity of staining and percentage of positive tumor cells. Staining intensities of 0, 1, 2, and 3 corresponded to negative, mild, moderate, and strong staining; percentages of positive tumor cells were estimated by the observers. The cutoff percentages of the cells for the dichotomization of data were determined for each staining individually by performing receiver operating characteristic (ROC) curve analyses. For *HIF-1α* and *Ki*-*67*, only nuclear staining was evaluated. Furthermore, for *EGFR*,*CAIX*, *c-Met*, *CLAUDIN-4*, and *GLUT1*, only cell membrane staining was evaluated. Both *VEGF* and *Bcl-2* showed a membranous or cytoplasmic staining pattern. *YAP-1* was visualized through cytoplasmic or nuclear staining. Representative images of immunohistochemistry are shown in Supplementary Appendix 6.

### HPV status determination by using p16

In this study, the overexpression of p16 detected through immunohistochemical staining was defined as a surrogate marker of HPV involvement. The expression of p16 was scored as positive in cases of strong and diffuse nuclear and cytoplasmic staining in ≥ 70% of tumor cells [4, 15]. Sixty patients (75%) were identified as having p16-negative PC; these patients were men with a median age of 51 years. The origin of the tumors was the oropharynx and hypopharynx in 33 and 27 patients, respectively. According to the American Joint Committee on Cancer (AJCC) staging, 4 and 56 had stage III and IV cancers, respectively. Table 1 shows the characteristics of the 60 patients.

### PET-CT image acquisition

All patients were examined using a PET-CT scanner (PET-CT-16 slice, Discovery STE; GE Medical System, Milwaukee, WI, USA) approximately 60 minutes after the administration of 370 MBq of ^18^F-FDG; they were required to fast for at least 4 hours before the scan. This procedure provided the standardized uptake value (SUV) of FDG; the maximum SUV (SUVmax) was confirmed by 2 nuclear medicine physicians.

### Measurement of metabolic tumor volume and total lesion glycolysis

The metabolic tumor volume (MTV) and total lesion glycolysis (TLG) were measured from attenuation-corrected FDG-PET images by using an SUV-based automated contouring program (Advantage Workstation Volume Share Version 2, GE Health). This procedure was described in our previous study [28]. The MTV was defined as the sum of the metabolic volumes of the primary tumors. We used an SUVmax of 2.5 (MTV2.5), an SUVmax of 3.0 (MTV3.0), 40% of SUVmax (MTV40%), and 50% of SUVmax (MTV50%). TLG was determined using the following formula: TLG = meanSUV × MTV. We used threshold levels equivalent with MTVs: TLG2.5, TLG3.0, TLG40%, and TLG50%. Two sets of TLG were determined for each patient: TLGp for the primary tumors and TLGw for the whole body. TLGw was calculated by summing TLGp and all other TLG values of metastatic neck lymph nodes.

### Delineation of CT-based tumor volume

The patients were simulated in an RT setup position on the table of a CT simulator with a head and neck immobilization device. The definition of CT-based tumor volume at the primary site (GTVp) was previously reported [31].

### Treatment and follow-up

RT was performed using a sequential intensity-modulated RT technique [31]. All patients received doses of 1.8 Gy daily and up to a total dose of 68.4–73.8 Gy to the primary tumors or metastatic lymph nodes. The median RT duration was 55 days. Forty-seven patients received concurrent chemotherapy with cisplatin (80–100 mg/m^2^ on days 1, 22, and 43). Ten patients received combined cetuximab (400 mg/m^2^ [loading dose] and 250 mg/m^2^) weekly because of being older than 70 years; 3 patients received RT alone.

After treatment completion, all patients were regularly followed. The definition of local failure was based on the laryngoscopy and neck CT results or both. If a patient had a persistent tumor or local recurrence after initial complete remission, salvage surgery was suggested, if technically feasible.

### Statistical analyses

This study used the median GTVp, SUVmax, MTV, and TLG values as cutoff values. To examine the correlations between the parameters and recurrence, ROC curves were constructed to identify the optimal predictive performance among MTVs, TLGs, and the scoring system for different genomic expressions patterns. Correlations between the volumetric and immunohistochemical data were examined using Pearson correlation, with the alpha level set at 0.01. The study endpoints were cause-specific survival (CSS), disease-free survival (DFS), and primary relapse-free survival (PRFS) rates, which were calculated using the Kaplan–Meier method and log-rank test. Multivariate analysis using Cox regression was performed to examine the effects of explanatory variables on CSS, DFS, and PRFS. Two-tailed tests were used, and *P* < .05 was considered statistically significant. All calculations were performed using SPSS 13.0 for Windows (SPSS Inc, Chicago, IL, USA).

## CONCLUSIONS

For patients with p16-negative advanced PCs requiring definitive RT or CRT, treatment outcomes can be stratified by the immunohistochemical biomarkers of *VEGF* and *GLUT1* and CT-based tumor volume. Further systematic or external validation studies would be required to verify our findings.

## SUPPLEMENTARY MATERIALS


