# Supplementary material for: Immunohistochemical biomarkers and volumetric parameters for predicting radiotherapy-based outcomes in patients with p16-negative pharyngeal cancer
Source: Oncotarget. 2017 Aug 21;8(42):72342–51. doi: 10.18632/oncotarget.20374 (PMC5641134; doi:10.18632/oncotarget.20374)
Supplement: Supplementary file 1 [file oncotarget-08-72342-s001.pdf]

Immunohistochemical biomarkers and volumetric parameters for predicting radiotherapy-based outcomes in patients with p16-negative pharyngeal cancer

SUPPLEMENTARY MATERIALS

Supplementary Appendix 1: The detailed patterns of treatment failure for the cohort (*N* = 60)

Outcome	*N* (%)
Initial response to RT (evaluate by the 1st image study after RT according the response evaluation criteria in solid tumors version 1.1	
Primary tumor response	
Complete response	38 (63%)
Partial response	22 (37%)
Neck LNs response	
Complete response	36 (60%)
Partial response	24 (40%
Status	
Alive	31 (52%)
Alive without disease	22
Alive with primary relapse	4
Alive with neck relapse	1
Alive with primary and neck relapse	4
Dead due to cancer recurrence	24 (40%)
Died of primary relapse	8
Died of neck relapse	1
Died of distant metastasis	2
Died of primary and neck relapse	10
Died of primary and distant relapse	1
Died of primary, neck and distant relapse	2
Died of other malignancies	5 (8%)

1. Cause-specific survival for patients who were staged with T1-2 and T3-4 (1A), and with N1 and N2-3 (1B) (P = 0.04 and P = 0.22, respectively).

1A

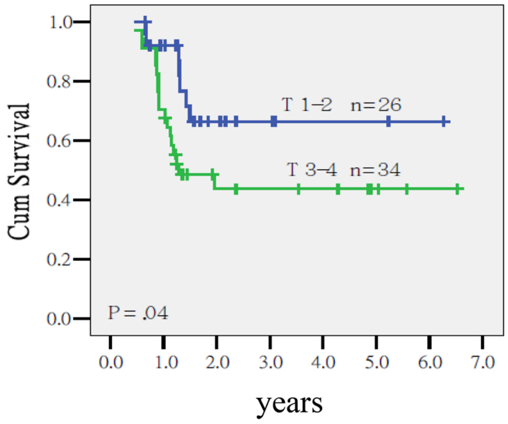

1B

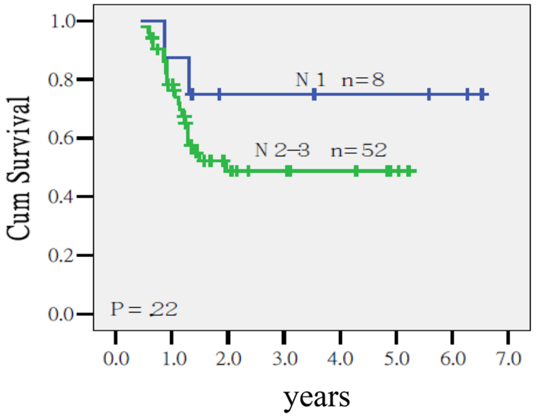

2. Disease-free survival for patients who were staged with T1-2 and T3-4 (2A), and with N1 and N2-3 (2B) (P = 0.18 and P = 0.08, respectively).

2A

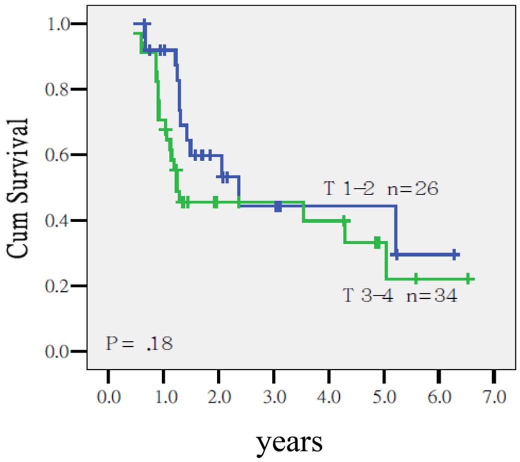

2B

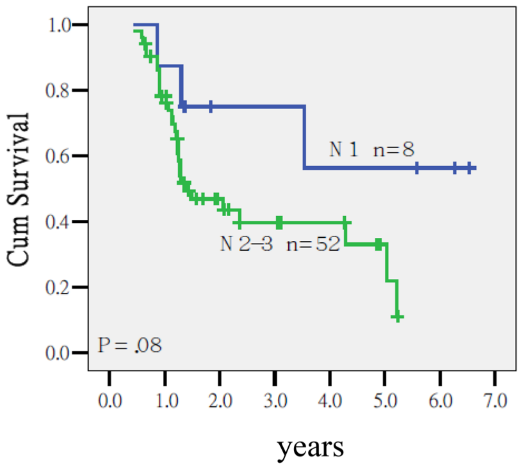

3. Primary relapse-free survival for patients who were staged with T1-2 and T3-4 (P = 0.37).

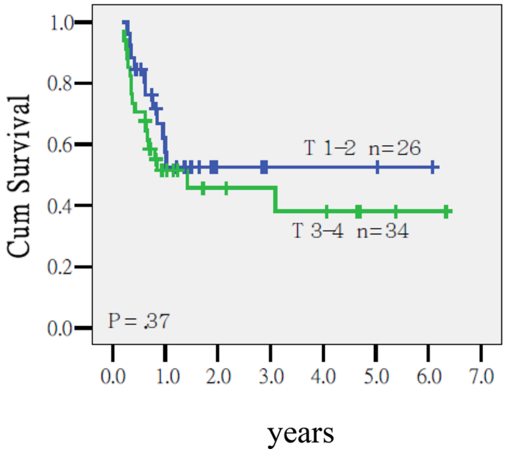

Supplementary Appendix 2: Survival curves according to *T*- and *N*-classification.

Supplementary Appendix 3: The value and ability of various threshold methods of PET-CT related parameters that predicted residual or recurrent tumor according to the continuous and the cut-offs using the median values

PET parameters	Median	Average ± SD (range)	Area under the curve	*p* value
SUVmax	10.3	10.5 ± 4.8 (1.9–24.1)	0.52 ± 0.08 (median)	0.24
			0.46 ± 0.08 (continuous)	0.32
MTV2.5 (ml)	14.1	29.0 ± 40.6 (0.1–281.0)	0.60 ± 0.07 (median)	0.19
			0.58 ± 0.08 (continuous)	0.38
TLGp40% (g)	62.4	20.9 ± 159.4 (7.9–1031.7)	0.63 ± 0.07 (median)	0.08
			0.59 ± 0.08 (continuous)	0.228
TLGw40% (g)	132.5	221.8 ± 231.3 (15.2–1058.8)	0.61 ± 0.07 (median)	0.13
			0.58 ± 0.08 (continuous)	0.28

Receiver operating characteristic curves were created to evaluate the predictive performance for tumor recurrence.

Supplementary Appendix 4: Receiver operating characteristic curves were created to evaluate the predictive performance for residual or recurrent tumor according to the continuous variables and the optimal cut-offs for immunohistochemical studies

**Variables**		***N* (%)**	**Area under the curve**	***p* value**
HIF-1α stain percentage (available cases = 57)				
	continuous	19 (33%) vs. 38 (67%)	0.59 ± 0.08	0.24
	< 80% vs. ≥ 80%		0.60 ± 0.08	0.20
*VEG*F (available cases = 59)				
	continuous	36 (61%) vs. 23 (39%)	0.72 ± 0.06	0.004
	IRS score 0-2vs. 3-12		0.66 ± 0.07	0.04
GLUT-1 stain percentage (available cases = 59)				
	continuous	29 (49%) vs. 30 (51%)	0.71 ± 0.06	0.002
	< 90% vs. ≥ 90%		0.67 ± 0.07	0.03
Claudin-4 (available cases = 57)				
	continuous	40 (70%) vs. 17 (30%)	0.58 ± 0.08	0.35
	IRS score 0–4 vs. 5–12		0.54 ± 0.08	0.62
CAIX stain percentage (available cases = 60)				
	continuous	37 (62%) vs. 23 (38%)	0.59 ± 0.07	0.27
	≦ 10% vs. > 10%		0.54 ± 0.07	0.62
c-Met stain intensity (available cases = 57)				
	continuous	29 (51%) vs. 28 (49%)	0.53 ± 0.08	0.66
	≦ 20% vs. > 20%		0.59 ± 0.08	0.25
Bcl-2 stain (available cases = 60)				
	continuous	42 (71%) vs. 17 (29%)	0.59 ± 0.08	0.29
	< 10% vs. ≥ 10%		0.57 ± 0.08	0.37
YAP-1 positive stain percentage (available cases = 59)				
	continuous	21 (36%) vs. 38 (64%)	0.53 ± 0.08	0.68
	< 50% vs. ≥ 50%		0.50 ± 0.08	0.96
Ki-67 stain percentage (available cases = 59)				
	continuous	30 (51%) vs.29 (49%)	0.62 ± 0.07	0.13
	< 15% vs. ≥ 15%		0.59 ± 0.08	0.37
EGFR stain percentage (available cases = 58)				
	continuous	32 (55%) vs. 26 (45%)	0.62 ± 0.07	0.09
	< 65% vs. ≥ 65%		0.59 ± 0.08	0.28

Supplementary Appendix 5: Multivariate analysis using the Cox regression model per sites for overall survival, disease-free survival, and primary relapse-free survival among volumetric parameters or protein biomarkers. See Supplementary Appendix 5.

Appendix 6

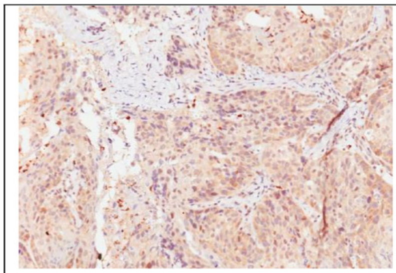	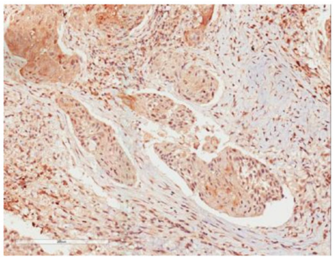	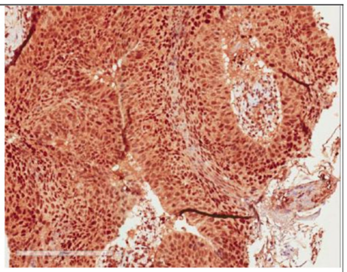
Negative HIF-1α staining (only non-specific cytoplasmic staining in the background)	Low HIF-1α staining	High HIF-1α staining

(A) Representative figures of nuclear staining for HIF 1α (Original magnification 200 X)

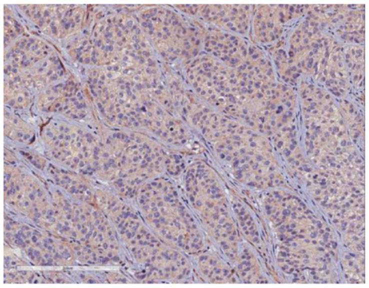	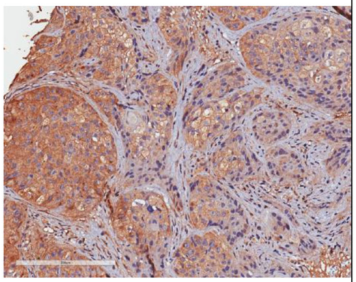	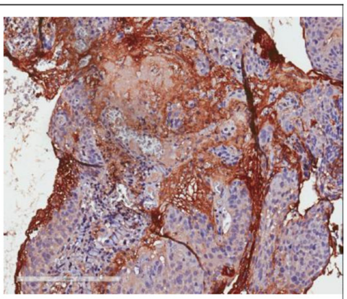
Weak VEGF staining	Moderate VEGF staining	strong VEGF staining

(B) Representative figures of VEGF staining with both membranous and/or cytoplasmic staining (Original magnification 200 X)

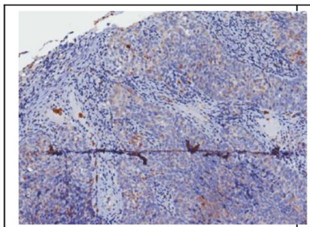	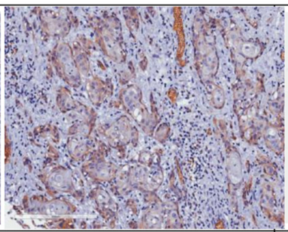	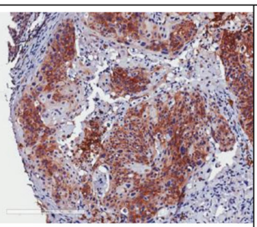	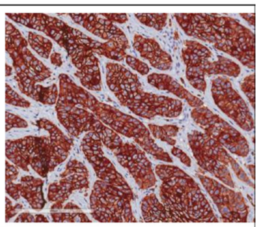
Negative GLUT-1 staining	Weak GLUT1 staining	Moderate GLUT1 staining	Strong GLUT1 staining

(C) Representative figures of membranous staining for GLUT1 (Original magnification 200 X)

Supplementary Appendix 6: IHC stain examples.
